# *SP7* gene silencing dampens bone marrow stromal cell hypertrophy, but it also dampens chondrogenesis

**DOI:** 10.1177/20417314231177136

**Published:** 2023-06-21

**Authors:** Rose Ann G Franco, Eamonn McKenna, Pamela G Robey, Ross W Crawford, Michael R Doran, Kathryn Futrega

**Affiliations:** 1 School of Mechanical, Medical and Process Engineering, Faculty of Engineering, Queensland University of Technology (QUT), Brisbane, QLD, Australia; 2 Translational Research Institute (TRI), Brisbane, QLD, Australia; 3 Center for Biomedical Technologies, Faculty of Engineering, Queensland University of Technology (QUT), Brisbane, QLD, Australia; 4 Skeletal Biology Section (SBS), National Institute of Dental and Craniofacial Research (NIDCR), National Institutes of Health (NIH), Department of Health and Human Services (DHHS), Bethesda, MD, USA; 5 School of Biomedical Sciences, Faculty of Health, Queensland University of Technology, Brisbane, QLD, Australia; 6 AstraZeneca, Biologics Engineering, Oncology R&D, One MedImmune Way, Gaithersburg, MD, USA

**Keywords:** *SP7 (Osterix)*, gene silencing, bone marrow stromal cells, mesenchymal stem cells, chondrogenesis, hypertrophy, osteogenesis, adipogenesis

## Abstract

For bone marrow stromal cells (BMSC) to be useful in cartilage repair their propensity for hypertrophic differentiation must be overcome. A single day of TGF-β1 stimulation activates intrinsic signaling cascades in BMSCs which subsequently drives both chondrogenic and hypertrophic differentiation. TGF-β1 stimulation upregulates *SP7*, a transcription factor known to contribute to hypertrophic differentiation, and *SP7* remains upregulated even if TGF-β1 is subsequently withdrawn from the chondrogenic induction medium. Herein, we stably transduced BMSCs to express an shRNA designed to silence *SP7*, and assess the capacity of *SP7* silencing to mitigate hypertrophy. *SP7* silencing dampened both hypertrophic and chondrogenic differentiation processes, resulting in diminished microtissue size, impaired glycosaminoglycan production and reduced chondrogenic and hypertrophic gene expression. Thus, while hypertrophic features were dampened by *SP7* silencing, chondrogenic differentation was also compromised. We further investigated the role of *SP7* in monolayer osteogenic and adipogenic cultures, finding that *SP7* silencing dampened characteristic mineralization and lipid vacuole formation, respectively. Overall, *SP7* silencing affects the trilineage differentiation of BMSCs, but is insufficient to decouple BMSC hypertrophy from chondrogenesis. These data highlight the challenge of promoting BMSC chondrogenesis whilst simultaneously reducing hypertrophy in cartilage tissue engineering strategies.

## Introduction

Bone marrow-derived stromal cells (BMSCs, sometimes referred to as mesenchymal stem cells) are an excellent cell source for cartilage tissue regeneration due to their abundant supply, ease of culture, and capacity to generate cartilage-like tissue.^1^ However, BMSCs do not yield a stable chondrocyte-like cell, and instead these cells have a propensity to undergo hypertrophic differentiation, contributing to a mineralized tissue unsuitable for use in cartilage defect repair. Several strategies have been proposed to minimize or prevent BMSC hypertrophy,^2^ but none have been widely replicated nor have these strategies yielded a viable clinical product. Recently, our group reported on the observation that BMSC respond to a single day of Transforming Growth Factor-β1 (TGF-β1) stimulation by engaging intrinsic signaling cascades which promote continued chondrogenic and hypertrophic differentiation even if TGF-β1 is subsequently removed from the medium.^3^ Immediately following TGF-β1 stimulation, *SP7* is upregulated, and it remains upregulated even if TGF-β1 is eliminated from the medium.

The *SP7*, also known as *Osterix*, gene codes for the zinc finger containing transcription factor SP7 (Osterix, Osx). In mesenchymal cells, *SP7* plays a major role in bone formation.^4^ When *SP7* is knocked out in mice, skull bones formed by intramembranous ossification do not mineralize and some skeletal elements formed by endochondral ossification suffer delayed mineralization.^5^ Postnatally, *SP7* contributes to bone growth and homeostasis.^6^ Knockout of *SP7* resulted in interrupted osteocyte maturation, altered osteocyte morphology and function, and reduction of osteoclast size and density.^6^

Our RNA-Seq data show that *SP7* is not expressed in human adult chondrocytes.^3^ Given that *SP7* is a transcription factor that appears to play a critical role in bone development, and that *SP7* expression is persistent in BMSCs following TGF-β1 stimulation, we reasoned that silencing *SP7* may be an effective strategy to mitigate BMSC hypertrophy. Previous studies which suggest *SP7* manipulation may enable control over hypertrophy include demonstration that siRNA *SP7* knockdown in MLB13MYC clone 17 cells upregulated the chondrogenic markers *Sox9*, *Col2*, and downregulated osteogenic markers *Col1*, *Alp* and *Bglap*.^7^ Silencing of *SP7* in ATDC5 chondroprogenitor cells inhibited the *Col10* expression,^8^ while *Sp7* over expression upregulated *Col10*, *Mp13*, *Alp*, and *Ibsp*.^9^

The role of *SP7* in the trilineage differentiation of BMSCs has not been previously demonstrated. The overall aim of this study was to determine if *SP7* silencing was sufficient to prevent BMSC hypertrophy during chondrogenesis, as well as to determine how *SP7* silencing influenced BMSC osteogenesis and adipogenesis. *SP7* silencing was achieved by transducing BMSC to constitutively express a short hairpin RNA (shRNA) engineered to target the *SP7* transcript. Transducing cells to express shRNA provides more stable control over the gene product than transfection with short interfering RNA (siRNA),^10^ and this approach can minimize the deleterious effects often associated with complete gene knockout methods.^11,12^ BMSC osteogenesis and adipogenesis was assessed in monolayer cultures, using readouts that included staining for mineral accumulation or lipid vacuoles, respectively, as well as gene expression. BMSC chondrogenesis and hypertrophy was assessed using a microtissue model, and a high-throughput microtissue culture platform, the Microwell-mesh.^13^ In previous work,^3^ we demonstrated that microtissues, compared to larger diameter pellet cultures, yield more homogenous tissue and that microtissues are a better tool for hypothesis testing and bioprocess optimization. Cartilage-like microtissues were characterized using readouts including glycosaminoglycan (GAG) quantification, immunohistochemistry, and gene expression.

## Materials and methods

### BMSC isolation and expansion

BMSCs were isolated as described previously.^13^ In brief, bone marrow aspirates were collected from consenting voluntary donors using protocols approved by the Mater Hospital Human Research Ethics Committee, and in accordance with the National Health and Medical Research Council of Australia guidelines (Ethics number: 1541A). Using Ficoll-Paque PLUS density gradient (GE Healthcare) centrifugation, mononuclear cells were enriched from 20 mL of bone marrow aspirate. The mononuclear cells were resuspended in low glucose Dulbecco’s Modified Eagle Medium (LG-DMEM), supplemented with 10% fetal bovine serum (FBS, ThermoFisher), 1% penicillin/streptomycin (PenStrep, Gibco), 10 ng/mL Fibroblast Growth Factor-1 (FGF-1, PeproTech), and 5 µg/mL Heparin (Sigma-Aldrich) as previously done in our laboratory.^13^ The cells were distributed into five T175 flasks (Nunc) with 35 mL of growth medium in each flask, and placed into a humidified, normoxic incubator (20% O_2_, 5% CO_2_, and 37°C). On the following day, the medium was exchanged to enrich for adherent cells, and the cells were grown in a hypoxic incubator (2% O_2_, 5% CO_2_, and 37°C) for further expansion. Media exchange was performed every 2–3 days until 80% confluence, and then cells were sub-cultured at 2 × 10^5^ cells per new T175 flask. BMSC were used up to passage 3 in subsequent experiments. Three BMSC donor cell populations were used in this study: Donor 1 was a 44-year-old male, Donor 2 was a 21-year old female, Donor 3 was a 24-year old male.

### Bacterial transformation and plasmid purification

A vial of One Shot^®^ Stbl3™ chemically competent *E. coli* cells (Thermo Fisher Scientific) was used for each transformation. A shRNA construct containing the target sequence for the *SP7* gene (AGGTGTATGGCAAGGCTTCGC ACCTGAAG) and a Scrambled Control were cloned in separate lentiviral GFP vectors with expression under the U6 promoter (pGFP-C-shLenti, Origene). Bacterial cells and plasmids were combined, incubated on ice, heat shocked at 42°C, and re-incubated on ice before inoculation in Luria Broth (LB) in a shaking incubator at 37°C. After the outgrowth step, transformants were screened by spread-plating 200 uL of suspension on Luria Agar plates with chloramphenicol (Sigma-Aldrich) (LA-C) and incubated at 37°C overnight. A single colony was streaked on LA-C plates and a purified colony was inoculated in 200 mL LB with chloramphenicol for overnight expansion in a shaking incubator at 37°C. Plasmids were isolated and purified using NucleoBond Xtra Midi Kit (Takara Bio) following the manufacturer’s protocol. Plasmid DNA concentration and purity were measured using a NanoDrop 1000 spectrophotometer (Thermo Fisher Scientific).

### Lentiviral production

To generate second-generation lentiviral particles, the following plasmids were used: psPAX2 for packaging and PMD2.G for VSV-G envelope expression (both from Addgene) combined with the shRNA expression plasmid and transfected into HEK293 FT (Thermo Fisher) at a 1:6 ratio with polyethylenimine (PEI, 2 mg/mL) in Opti-MEM culture medium (Gibco). Medium was replaced after 18 h, and viral particles were harvested 48 h post-transfection. Viral particle containing supernatant was collected after centrifugation and concentrated using Lenti-X Concentrator (Takara Bio) following the manufacturer’s protocol. Viral titre was determined based on GFP expression using flow cytometry (BD LSR Fortessa X20).

### BMSC transduction with lentivirus

BMSC transduction was performed following a protocol reported by Lin et al. with minor modifications.^14^ In brief, BMSCs were seeded in 6-well plates (5 × 10^4^ cells/ well) and allowed to expand. Cell number was determined and transduced with an MOI of 10 with a final volume of 1 mL. Medium was replaced after 24 h, and cells were passaged upon confluence. Transduced cells were sorted based on GFP expression (BD Astrios Sorter) and then expanded in culture as described above. Only cells up to two passages post-sorting were used in the succeeding experiments. ScControl corresponds to BMSCs transduced with Scrambled Control-containing shRNA, while shRNA-*SP7* corresponds to BMSCs transduced with shRNA specific for *SP7* silencing. Wild-type (WT) corresponds to non-transduced BMSC controls.

### Microwell-mesh fabrication and use

The Microwell-mesh is a high throughput microtissue culture platform, described previously.^13^ Small diameter microtissues yield more homogenous tissue, relative to conventional pellet cultures,^13,15^ and the Microwell-mesh enables efficient culture of hundreds of uniform microtissues, in a manner ideal for studying BMSC chondrogenic differentiation.^3,16^ In brief, the Microwell-mesh base was fabricated from a sheet of polydimethylsiloxane (PDMS, SYLGARD™ 184 Silicone Elastomer Kit) which had been cast on a surface having features which were the negative of the microwell pattern. The microwell dimensions were 2 mm × 2 mm square, by 0.6 mm deep. The PDMS sheets were shaped into discs to fit into well plates using a wad punch (Amazon.com). Nylon (6/6) mesh having 36 × 36 µm openings was bonded over the microwell opening using silicone glue (Selley’s Aquarium Safe). The discs were then anchored into well plates with a dab of silicone glue. The Microwell-mesh, and the plate containing the inserts, was sterilized by first placing ~3 mL of 80% ethanol into each well, and centrifuging the plate at 3000 ×*g* for 5 min. This high-speed spin displaced air bubbles from the microwells and forced ethanol to contact all surfaces in and under the Microwell-mesh insert. The plates were then fully submerged in 80% ethanol for at least 1 h. Wells were then washed twice with Dulbecco’s phosphate-buffered saline (PBS) and once with water (Invitrogen UltraPure™ DNase/RNase-Free distilled water). Prior to use in cell culture, to make the PDMS surface non adhesive and promote cell aggregation,^17,18^ the Microwell-mesh inserts were coated with Pluronic. Each well was filled with 3 mL of Pluronic solution (5% Pluronic-F127 in DPBS w/v; Sigma-Aldrich) and the plate was centrifuged 3000×*g* for 5 min to force the solution into the microwells and displace bubbles from microwells. Wells were washed once with PBS before seeding with BMSC.

### Differentiation assays

To assess the chondrogenic potential of transduced BMSCs, cells were seeded into 12-well Microwell-mesh plates at a density of 0.5 × 10^6^ BMSCs/well (~5000 BMSCs/microwell) in chondrogenic induction medium. Chondrogenic induction medium was formulated from high glucose (HG)-DMEM supplemented with GlutaMAX, 1% PenStrep, 1% Sodium Pyruvate, 1% Insulin-Transferrin-Selenium-Ethanolamine Solution (ITS-X), 40 µg/mL L-Proline (Sigma-Aldrich), 200 µM L-ascorbic acid-2-phosphate (Sigma-Aldrich), 100 nM Dexamethasone (Sigma-Aldrich) and 10 ng/mL TGF-β1 (PeproTech). To pellet cells and promote cell aggregation, the Microwell-mesh plates were centrifuged at 400× *g* for 3 min. Cultures were then incubated for 14 days at 2% O_2_, 5% CO_2_, and 37°C with medium exchanges performed every 2 days. To monitor the growth of chondrogenic microtissues, microscope images were captured at day 2, 4, 6, 8, 10, 12, and 14 of culture using an Olympus BX61/ IX73 microscope and size was determined using ImageJ. Stable transduction of chondrogenic microtissues was monitored and validated by capturing fluorescent images of the transduced cells which all carried a GFP reporter as well as the scrambled or *SP7* shRNA.

To assess the osteogenic and adipogenic potential, BMSCs were seeded at 3 × 10^4^ cells/cm^2^ in 12-well plates for RNA collection (Nunc) and 48-well plates (Nunc) for staining assays and calcium content quantification. Cultures were maintained in osteogenic induction medium, formulated from HG-DMEM supplemented with 10% FBS, 1% PenStrep, 10 mM β-glycerol phosphate (BGP, Sigma-Aldrich), 100 nM Dexamethasone, and 50 µM L-ascorbic acid-2-phosphate, 100 ng/mL BMP-2 (Medtronic)) and adipogenic induction medium (HG-DMEM, 10% FBS, 1% PenStrep, 1 µg/ mL Insulin (Gibco), 100 nM Dexamethasone, 200 µM Indomethacin (Sigma-Aldrich), 500 µM 3-isobutyl-1-methylxanthine (IBMX; Sigma-Aldrich)). Cultures were maintained for 14 days, and medium was exchanged every 3 days.

### Characterization of chondrogenic cultures

GAG and DNA were quantified as described previously.^13^ Briefly, microtissues were digested in 125 µg/mL of papain and 10 mM L-cysteine (Sigma-Aldrich) in 100 mM of PBE buffer at pH 6.5, overnight at 60 ºC. DNA in the digest was quantified using a Quant-iT PicoGreen dsDNA assay Kit (Thermo Fisher Scientific), read in a fluorescent microplate reader (FLUOstar Omega) at 485 nm excitation and 520 nm emission.^19^ Quantification of GAG in the papain-digested samples was performed using the dimethylmethylene blue (DMMB; Sigma-Aldrich) assay. The plate was read using a microplate reader at 540 nm (Multiskan Go; Thermoscientific) and GAG was estimated using a standard curve of serial dilutions of chondroitin sulfate derived from shark cartilage (Sigma-Aldrich). For both DNA and GAG assays, the reported values were the average of four biological replicate well (12-well) cultures.

At harvest, microtissues were washed with PBS and fixed overnight in 4% paraformaldehyde (PFA, Sigma -Aldrich). Fixed samples were embedded in Tissue-Tek optimal cutting temperature compound (OCT, Sakura Finetek) and stored at −20°C. Cryosectioning was performed using a Leica Cryostat CM 1950, and 7 µm sections were collected on poly-lysine coated slides (ThermoFisher). Tissue sections were gently washed with PBS to remove OCT, slides fixed in 4% paraformaldehyde (PFA) for 30 min, and then stained with Alcian Blue (Sigma-Aldrich).

For immunohistological staining of specific collagens, tissue sections were treated with 2 mg/mL hyaluronidase (Sigma-Aldrich), permeabilized with 0.1% Triton X-100 (Sigma-Aldrich) and blocked with 10% normal goat serum (Thermo Fisher Scientific). Sections were stained with the following primary antibodies (Abcam): anti-collagen type II (ab34712) and anti-collagen type X (ab58632). Primary antibodies were suspended in 1% bovine serum albumin (BSA, Sigma-Aldrich) and incubated on tissue sections overnight at 4°C. The secondary antibody (goat anti-rabbit IgG-HRP; ab6721, Abcam) was applied the following day at room temperature for 1 h, and the DAB chromogen kit (Abcam) was used to stain the sections following the manufacturer’s protocol. Tissue sections were counterstained with nuclear fast red (Sigma-Aldrich) for 5 min and washed repeatedly. Slides were dehydrated and coverslipped using Eukitt mounting medium, and sections were imaged with an Olympus IX73 microscope.

### Characterization of osteogenic and adipogenic cultures

After 14 days of osteogenic and adipogenic induction culture, the relative metabolic activity was measured using the alamarBlue assay (Thermo Fisher Scientific). Cultures were incubated for 3 h at 37°C and read using a fluorescence spectrophotometer (FLUOstar Omega) using a spectral excitation/emission of 544 and 590 nm.

Following the specified days of culture, monolayers were fixed with 4% PFA for 30 min and then stained with Alizarin Red S (Sigma-Aldrich) for 30 min at room temperature. Excess stain was repeatedly washed out with distilled water. Brightfield images were captured using an Olympus IX73 microscope. Calcium was quantified using the O-Cresophthalein (OCPC) assay.

Osteogenic and adipogenic cultures were washed with PBS, fixed with 4% PFA for 30 min, and stained with Oil Red O (ORO) stain (Sigma-Aldrich) for 15 min. Excess stain was washed out with distilled water. Estimation of area occupied by lipid vacuoles in culture wells was performed using image analysis (ImageJ) of multiple regions in each replicate well, from each BMSC donor (*n* = 20). Brightfield mages were captured with Olympus IX73 microscope. Osteogenic and adipogenic cultures stained with ORO were also counterstained with DAPI nuclear stain and imaged with an Olympus IX73 microscope. Appropriate fluorescent filters were used for DAPI to view the nucleus, GFP to monitor stable transduction after 14 days, and ORO to view lipid vacuoles in GFP-expressing cells. Merged images were generated using CellSens imaging software (Olympus).

### Gene expression quantification using qRT-PCR

Total RNA was isolated using the ISOLATE II RNA Mini Kit (Bioline), following the manufacturer’s instructions. Cell lysis was performed using the accompanying RLY lysis buffer. Chondrogenic microtissues were crushed, to liberate RNA, in microcentrifuge tubes using micropestles (Sigma-Aldrich). Samples were treated on-column with the DNase-I included in the kit in accordance with the manufacturer’s protocol. RNA concentration and purity were measured using a NanoDrop 1000 spectrophotometer (Thermoscientific).

Extracted RNA was reverse-transcribed using SensiFAST cDNA Synthesis Kit (Bioline) to produce cDNA. Synthesized cDNA was combined with forward and reverse primers (200 nM), and SYBR Green PCR Master Mix (Applied Biosystems) and analyzed on a Viia7 Real Time (RT)PCR System (Applied Biosystems). Primer sequences are detailed in Supplementary Table 1. *GAPDH* all was used as housekeeping gene for samples. The run parameters were as follows: a single initial cycle of 50°C for 2 min and 95°C for 10 min, followed by 40 cycles of 95°C for 15 s and 60°C for 1 min. Target gene expression relative to housekeeping gene expression was calculated using the formula, 2^(Ct(Gene of interest) – Ct(*GAPDH*)).

Initial characterization of *SP7* was performed with quantitative (q) RT-PCR for all non-transduced BMSC donor cultures (WT) and the following conditions included compared: (1) For chondrogenic induction, Day 0 samples included expanded BMSCs prior to induction, Non-induced monolayer samples were grown in DMEM-HG supplemented with 10% FBS and 1% PenStrep for 14 days. No TGF-β1 and plus TGF-β1 controls were cultured in the Microwell-mesh in chondrogenic medium without or with TGF-β1, respectively; (2) For osteogenic induction, Day 0 samples, Non-induced samples and induced samples. Non-induced monolayer samples were cultured in DMEM-HG supplemented with 10% FBS and 1% PenStrep for 14 days, no BMP-2 samples were maintained in osteogenic medium without BMP-2 for 14 days, and BMP-2 samples were cultured in osteogenic medium supplemented with BMP-2; (3) For adipogenic induction, Day 0, Non-induced samples were compared with BMSC monolayer cultures maintained in adipogenic medium for 14 days.

### Statistical analysis

Samples were analyzed for normal distribution using the Shapiro-Wilk test (α = 0.05). For samples that were normally distributed, one-way analysis of variance (ANOVA) and Dunnett’s multiple comparison test were performed for comparison of three or more groups, and unpaired *t*-test for two groups. For samples that were not normally distributed, analysis was done using the Kruskal Wallis test and Dunn’s multiple comparison test for three or more groups, and Mann-Whitney test for comparison of two data sets. All statistical analyses were done in Graph Pad Version 9.2.0. A *p*-value of less than 0.05 was considered significant. Values represent the mean of four replicate well cultures for each BMSC donor unless otherwise stated ± the standard deviation (SD). For image analysis of lipid vacuoles in osteogenic and adipogenic cultures, 20 frames were analyzed for each condition for all three BMSC donors.

## Results

### SP7/ Osx is highly expressed in BMSC chondrogenic cultures with TGF-β1 and osteogenic cultures with BMP-2

*SP7* expression was initially quantified in non-transduced BMSCs (WT) to determine the conditions when *SP7* is upregulated. BMSCs were induced using chondrogenic, osteogenic, and adipogenic conditions for 14 days and compared to Day 0 and 14-day BMSC non-induced cultures. *SP7* gene expression significantly increased when chondrogenic medium was supplemented with TGF-β1 (10 ng/mL), relative to Day 0 and non-induced monolayer cultures, or chondrogenic microtissues without TGF-β1 for all BMSC donors (Supplemental Figure S1a).

BMSC osteogenic cultures supplemented with BMP-2 (100 ng/mL) showed significant upregulation of *SP7* gene expression compared to Day 0 and non-induced monolayer cultures, as well as osteogenic cultures without BMP-2 (Supplemental Figure S1b). There was no significant increase in *SP7* gene expression in BMSC adipogenic induction cultures relative to Day 0 and non-induced monolayer cultures for all three BMSC donors after 14 days of culture (Supplemental Figure S1c).

### Effect of SP7/Osx gene silencing on the growth of BMSC chondrogenic microtissues

**Figure 1.**
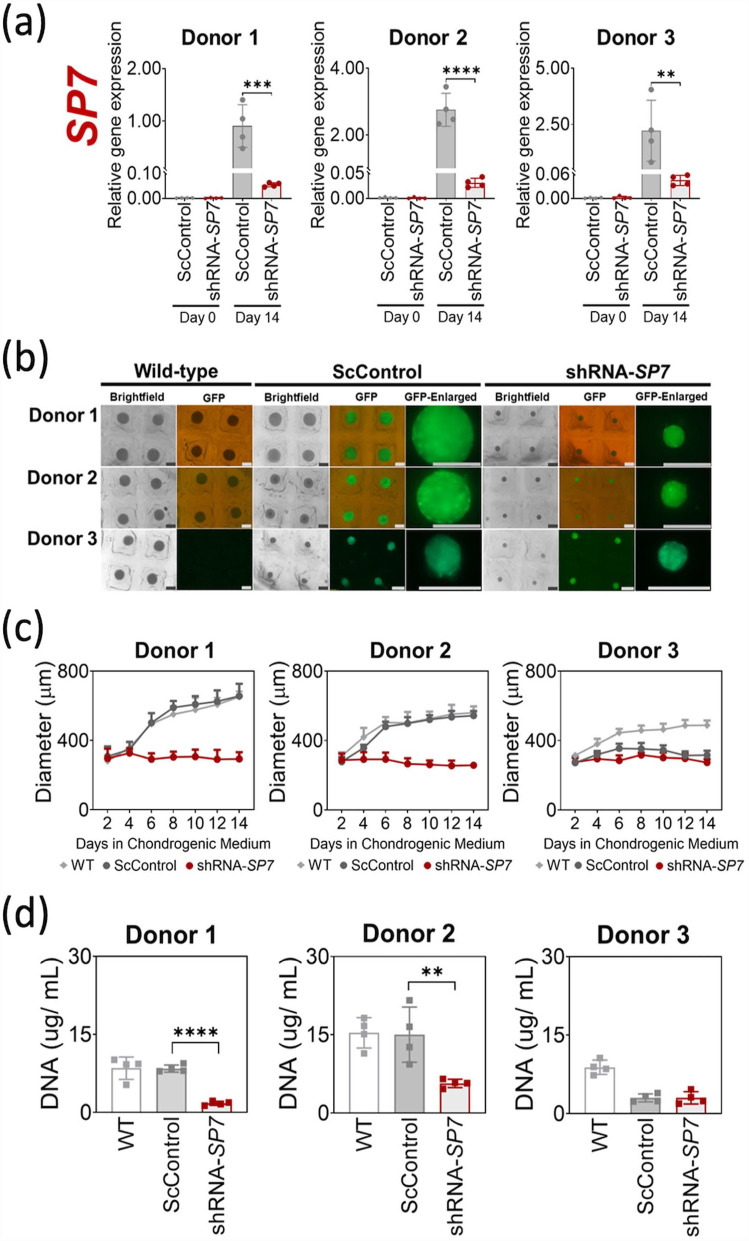
*SP7* silencing reduced microtissue growth during BMSC chondrogenic induction. (a) Silencing of *SP7* was verified by qRT-PCR after 14 days of chondrogenic induction with TGF-β1 (10 ng/mL). Significant reduction of *SP7* gene expression was observed in shRNA-*SP7* microtissues compared to ScControl for all three BMSC donors. Day 0 corresponds to expanded monolayer cultures of transduced BMSCs prior to chondrogenic induction. (b) Microscopic images of BMSC chondrogenic microtissues after 14 days of culture showing reduced size of shRNA-*SP7* microtissues for all donors. GFP was monitored using fluorescence microscopy to validate stable transduction after chondrogenic induction for both ScControl and shRNA-*SP7* microtissues and absence in WT microtissues. (c) Measurement of microtissue diameter through image analysis (*n* = 16) every 2 days during 14 days of chondrogenic culture. (d) DNA content measured through PicoGreen assay after 14 days of culture. Donor 1 and Donor 2 showed significant reduction of DNA in *SP7*-silenced cultures compared to WT and ScControl (*n* = 16). Shapiro-Wilk test was done to test for normal distribution (α = 0.05). One-way ANOVA and Dunnett’s multiple comparison test were done for normally distributed samples. Kruskal Wallis test and Dunn’s multiple comparisons test were done on samples that were not normally distributed. Statistical significance was indicated only in transduced cultures (ScControl and shRNA-*SP7*). Data plots represent the means of 4 replicate wells unless otherwise stated. *p < 0.05, **p < 0.01, ***p < 0.001, and ****p < 0.0001.

Silencing of the *SP7* gene in transduced BMSC microtissues was validated using qRT-PCR after 14 days of chondrogenic induction with TGF-β1 (Figure 1(a)). Similar to the behavior exhibited by WT BMSCs (Supplemental Figure S1a), Day 0 monolayer cultures of ScControl and shRNA-*SP7* showed insignificant to undetectable *SP7* gene expression (Figure 1(a)). *SP7* gene expression was upregulated when BMSCs were chondrogenically induced with TGF-β1 (ScControl), but downregulated when this was performed using cells with *SP7* silenced (shRNA-*SP7*) for all BMSC donors (Figure 1(a)).

GFP fluorescence was monitored in BMSC chondrogenic microtissues throughout the 14-day culture period as an indicator of stable transduction. GFP fluorescence was observed in ScControl and shRNA-*SP7* microtissue cultures for all donors over the 14-day cultures (Figure 1(b)). Non-transduced control microtissues (WT) did not exhibit fluorescence under the same microscope settings, demonstrating that fluorescent signal in other cultures was not due to autofluorescence (Figure 1(b)). shRNA-*SP7* microtissues were noticeably smaller in size compared to WT and ScControl microtissues for two out of three BMSC donors (Figure 1(b)).

The average diameters of WT and ScControl microtissues increased from day 2 to day 14 for all BMSC donors, with similar growth patterns observed for Donor 1 and 2 microtissues (Figure 1(c)). In all BMSC donors, the shRNA-*SP7* microtissues did not significantly increase in size from day 2 to day 14 (Figure 1(c)). At endpoint, the average microtissue diameters were as follows: Donor 1 (WT = 650.9 µm, ScControl = 655.9 µm, shRNA-SP7 = 293.4 µm); Donor 2 (WT = 559.7 µm, ScControl = 543.2 µm, shRNA-SP7 = 257.6 µm); and Donor 3 (WT = 487.1 µm, ScControl = 315.4 µm, shRNA-SP7 = 273.0 µm).

The DNA content of the microtissues per well was quantified at day 14. When the size of microtissues was compared to the DNA content, the size reduction correlated with the quantity of DNA recovered. The DNA content was significantly lower in shRNA*-SP7* microtissues compared to WT and ScControl for Donors 1 and 2, while there was no significant difference observed between ScControl and shRNA-*SP7* for Donor 3 (Figure 1(d)).

### Chondrogenesis was abrogated with SP7/ Osx gene silencing

**Figure 2.**
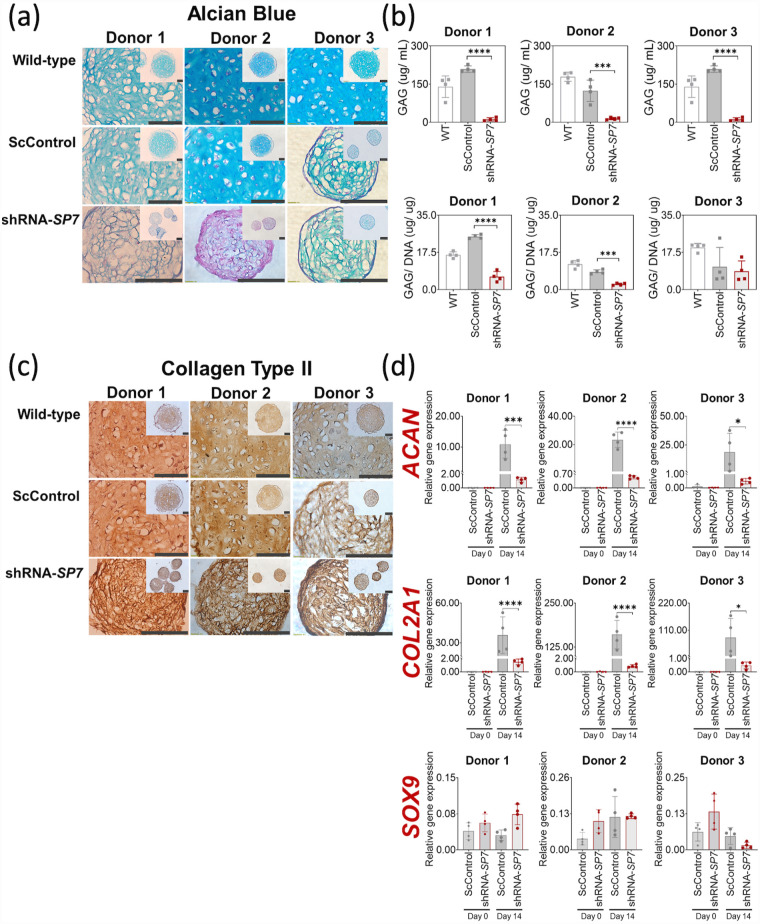
Chondrogenesis was abrogated with *SP7* silencing. (a) Alcian Blue staining of chondrogenic microtissues after 14 days of culture where GAG in Donor 1 and 2 is reduced with *SP7* silencing. Scale bar = 100 µm. (b) GAG and DNA content was measured after 14 days of culture. GAG and GAG/ DNA were significantly reduced with *SP7*-silencing in 2/3 donors, compared with shRNA-*SP7*. (c) The presence of collagen type II was detected in WT, ScControl and *SP7*-silenced chondrogenic microtissues after 14 days of culture. Scale bar = 100 µm. (d) Gene expression of chondrogenic markers were quantified using qRT-PCR. *ACAN* and *COL2A1* were reduced in all three donors. Shapiro-Wilk test was done to test for normal distribution (α = 0.05). One-way ANOVA and Dunnett’s multiple comparison test were done for normally distributed samples. Kruskal Wallis test and Dunn’s multiple comparisons test were done on samples that were not normally distributed. Statistical significance was indicated only in transduced cultures (ScControl and shRNA-*SP7*). Data plots represent the means of 4 replicate wells unless otherwise stated. *p < 0.05, **p < 0.01, ***p < 0.001, and ****p < 0.0001.

To determine the impact of *SP7* silencing on BMSC chondrogenesis, Alcian blue staining was performed to detect the GAG matrix content following 14 days of chondrogenic induction with TGF-β1. GAG staining was visibly reduced in shRNA-*SP7* microtissues for all BMSC donors, as was the formation of lacunae for Donor 2 BMSC (Figure 2(a)). For Donor 3, GAG staining and lacunae morphology were similar between ScControl and shRNA-*SP7* (Figure 2(a)). When GAG was quantified, shRNA-*SP7* microtissues showed significantly reduced production relative to WT and ScControls for two out of three donors (Figure 2(b)). The pattern of reduced GAG production was found to be similar for all BMSC donors when GAG was normalized to DNA content (Figure 2(b)).

Collagen type II is one of the primary matrix molecules in cartilage and is commonly used as a marker of BMSC chondrogenesis. Collagen type II was detected in all BMSC microtissue samples, including the shRNA-*SP7* microtissues (Figure 2(c)). However, the morphology of shRNA-*SP7* microtissues for two out of three BMSC donors was considered inferior to WT and ScControl microtissues where lacunae, characteristic of mature cartilage, were more prevalent (Figure 2(c)).

Chondrogenic gene markers, *ACAN* and *COL2A1*, were upregulated after 14-day chondrogenic induction in WT and ScControl microtissues, but were significantly downregulated in shRNA-*SP7* microtissues for all BMSC donors (Figure 2(d)). In contrast, the gene silencing did not generally affect *SOX9* gene expression (Figure 2(d)).

### Hypertrophy persists after SP7/ Osx gene silencing in BMSC chondrogenic microtissues

**Figure 3.**
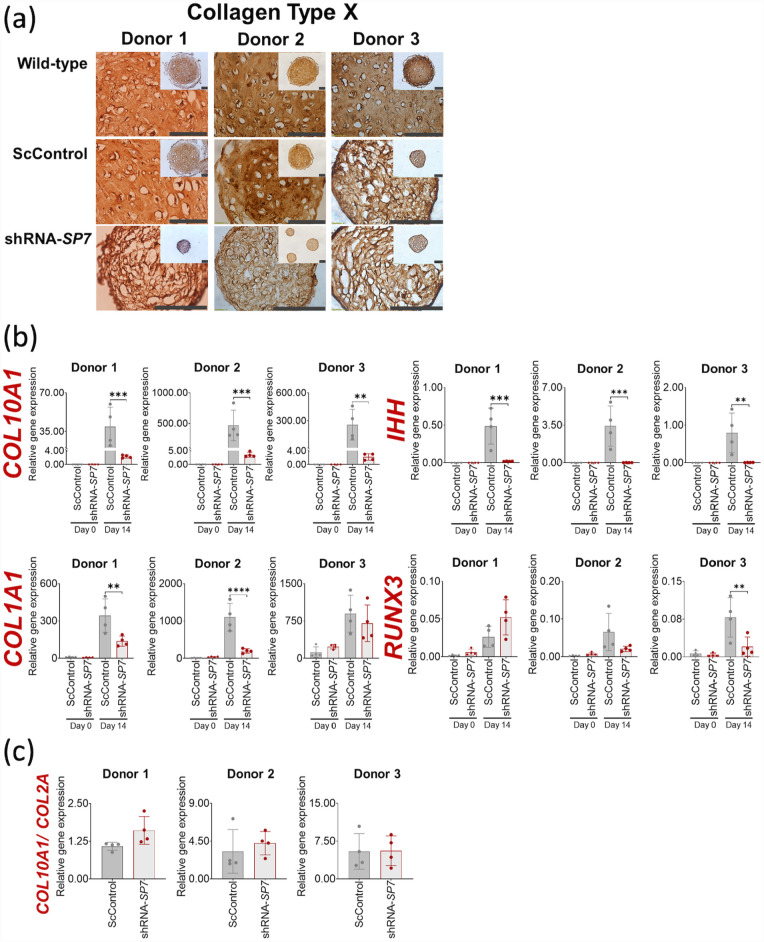
*SP7* silencing reduced expression of hypertrophic gene markers in BMSC chondrogenic cultures. (a) Collagen type X staining of BMSC chondrogenic microtissues after 14 days of culture. (b) Gene expression of hypertrophic markers using qRT-PCR. Silencing of *SP7* showed reduction of *COL10A1* after 14 days of chondrogenic induction in all three donors. *COL1A1* gene expression was reduced in 2/3 donors, while *RUNX3* was not affected by *SP7* gene silencing. (c) *SP7* gene silencing did not significantly reduce the pro-hypertrophic properties indicated by the *COL10A1*/*COL2A1* ratio in all three BMSC donors. Shapiro-Wilk test was done to test for normal distribution (α = 0.05). One-way ANOVA and Dunnett’s multiple comparison test were done for normally distributed samples. Kruskal Wallis test and Dunn’s multiple comparisons test were done on samples that were not normally distributed. Statistical significance was indicated only in transduced cultures (ScControl and shRNA-*SP7*). Data plots represent the means of 4 replicate wells. *p < 0.05, **p < 0.01, ***p < 0.001, and ****p < 0.0001.

Silencing of *SP7* did not visibly reduce the production of collagen type X in shRNA-*SP7* chondrogenic microtissues (Figure 3(a)). The presence of this protein suggested that *SP7* silencing did not prevent the hypertrophy in chondrogenic microtissues for all BMSC donors. Nevertheless, while there was visible collagen type X staining in all tissues, *COL10A1* gene expression was consistently reduced in all shRNA-*SP7* microtissues relative to ScControls (Figure 3(b)). In a similar manner, downregulation of *IHH* was also evident when *SP7* was silenced in microtissues from all BMSC donors compared to ScControl (Figure 3(b)). Downregulation of *COL1A1* in *SP7*-silenced microtissues was only observed for Donors 1 and 2; while *RUNX3* gene expression was unaffected in these two donors (Figure 3(b)). When *COL10A1* was normalized to *COL2A1*, shRNA-*SP7* microtissues were not significantly different from ScControl for all BMSC donors (Figure 3(c)). This indicates that *COL10A1* was downregulated with *COL2A1* expression during *SP7* gene silencing.

### Effect of SP7/Osx gene silencing on BMSC osteogenic cultures

**Figure 4.**
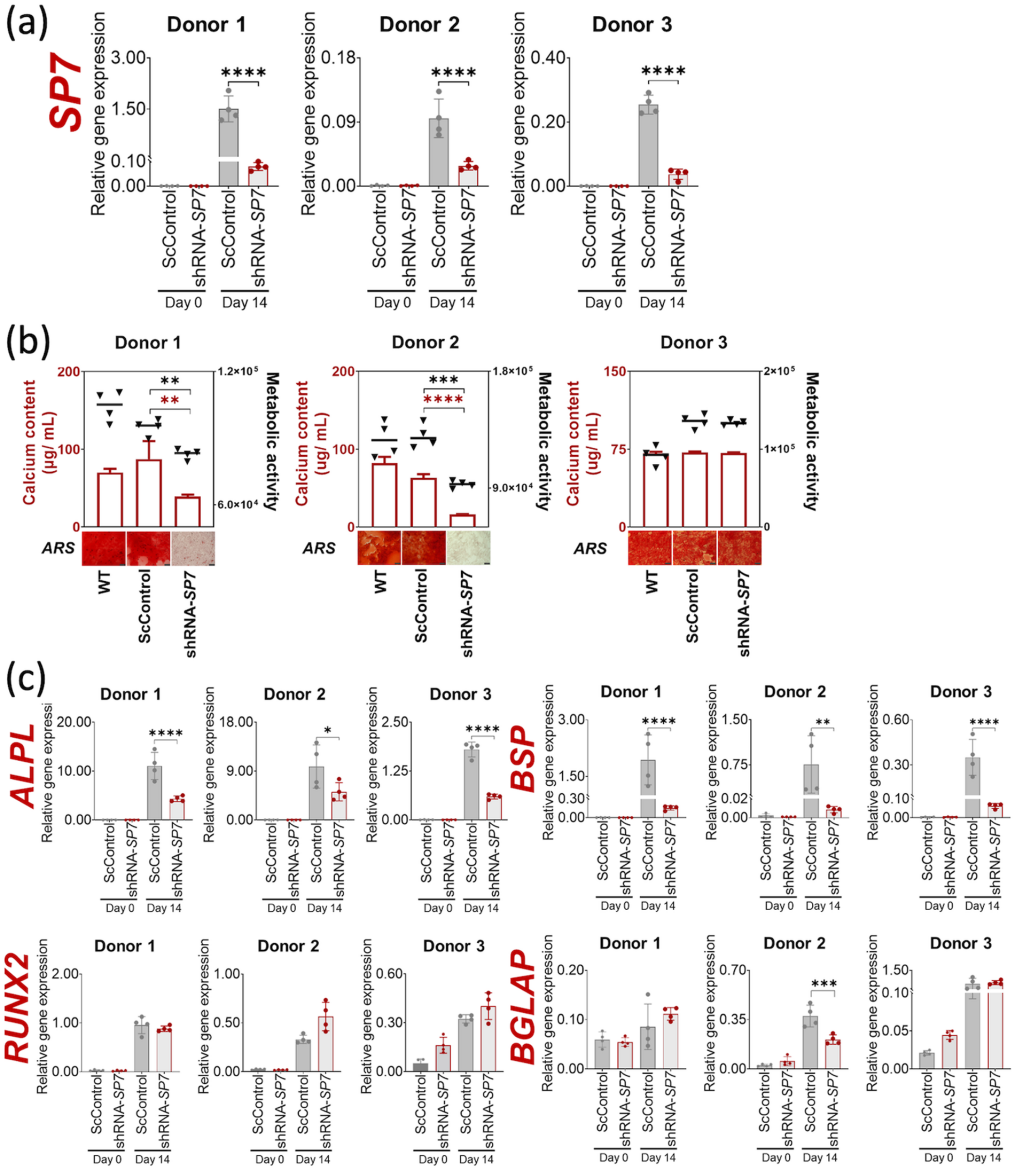
Effects of SP7 gene silencing on mineralization of BMSC osteogenic cultures with BMP-2 (100 ng/ mL). (a) Silencing of *SP7* gene was verified by qRT-PCR after 14 days of osteogenic induction with BMP-2 (100 ng/mL). A significant reduction in *SP7* gene expression was observed in shRNA-*SP7* samples for all three BMSC donors. (b) Calcium quantification showed a significant reduction in calcium deposition in shRNA-*SP7* samples compared to ScControl for Donor 1 and Donor 2. Relative cell viability measured using the alamarBlue assay showed significantly reduced cell number at culture endpoint in shRNA-*SP7* samples compared to ScControl in Donor 1 and Donor 2. Microscopic images of osteogenic cultures stained with Alizarin Red S (ARS) showing bright red color, indicative of mineralization (See Supplemental Figure S2 for ARS staining of whole plates). Scale bar = 100 µm. (c) Osteogenic gene expression markers, *ALPL* and *BSP*, were significantly downregulated by *SP7* silencing, while *RUNX2* and *BGLAP* were unaffected. Shapiro-Wilk test was done to test for normal distribution (α = 0.05). One-way ANOVA and Dunnett’s multiple comparison test were done for normally distributed samples. Kruskal Wallis test and Dunn’s multiple comparisons test were done on samples that were not normally distributed. Statistical significance was indicated only in transduced cultures (ScControl and shRNA-*SP7*). Data plots represent the means of 4 replicate wells unless otherwise stated. *p < 0.05, **p < 0.01, ***p < 0.001, and ****p < 0.0001.

Day 0 monolayer ScControl and shRNA-*SP7* cultures had minimal *SP7* gene expression prior to osteogenic induction for all BMSC donors (Figure 4(a)). Validation of *SP7* gene silencing in transduced BMSC osteogenic cultures was performed using qRT-PCR following 14 days of culture with BMP-2 (100 ng/mL). A significant reduction in *SP7* gene expression was consistently observed in osteogenically induced shRNA-*SP7* cultures for all BMSC donors, relative to ScControl under the same culture conditions (Figure 4(a)).

Metabolic activity, measured using the alamarBlue assay, indicated that shRNA-*SP7* cells were significantly less metabolically active than ScControl cells generated from Donors 1 and 2 (Figure 4(b)). Consistent with reduced metabolic activity, there was a reduction in the calcium content of shRNA-*SP7* cultures relative to ScControls for two out of three donors (Figure 4(b)). BMSC osteogenic cultures were stained with Alizarin Red S to highlight mineral deposits. There was little Alizarin Red S staining for monolayer cultures generated from Donors 1 and 2 shRNA-*SP7* cells, consistent with the levels of calcium measured by the OCPC assay (Figure 4(b) and Supplemental Figure S2). Donor 3 shRNA-*SP7* samples showed positive Alizarin Red S staining, comparable to WT and ScControl samples (Figure 4(b) and Supplemental Figure S2).

BMSC osteogenic cultures were assessed on day 0 and day 14 to determine the effect of *SP7* gene silencing on osteogenic gene expression markers. Insignificant *ALPL* and *BSP* gene expression was observed in day 0 cultures for all BMSC donors and while these were upregulated in 14-day osteogenic culture, silencing of *SP7* gene was observed to reduce this expression, even in the presence of BMP-2 (Figure 4(c)). No significant changes were seen in the gene expression, of *RUNX2* and *BGLAP* in osteogenic cultures, except a downregulation of *BGLAP* in Donor 2 (Figure 4(c)).

**Figure 5.**
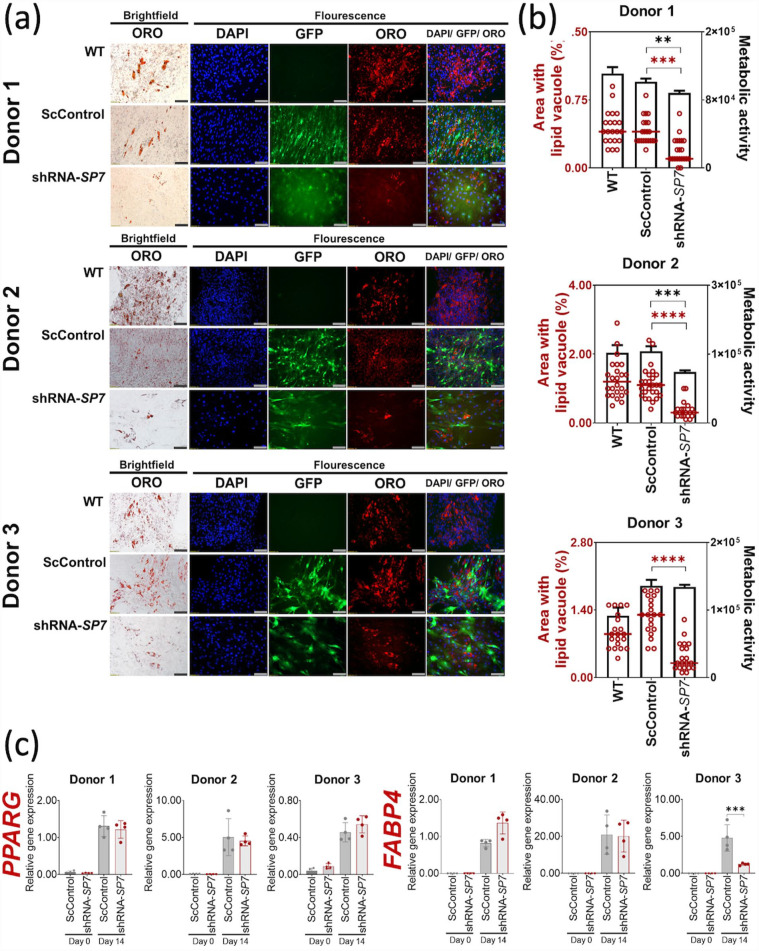
Effects of SP7 gene silencing on adipogenesis of BMSC osteogenic cultures with BMP-2 (100 ng/ mL). (a) Lipid vacuoles stained with Oil Red O (ORO, red) were visibly reduced in shRNA-*SP7* cultures in all BMSC donors. Fluorescence imaging shows the presence of DAPI-stained nucleui (blue) and GFP (green) (in ScControl and shRNA-*SP7* samples), indicating stable transduction after 14 days of culture. Scale bar = 100 µm. (b) Quantification of ORO-stained lipid vacuoles was measured using ImageJ (*n* = 20). A significant reduction of lipid vacuole formation was observed in shRNA-*SP7* cultures in all three BMSC donors. (c) Adipogenic gene markers were quantified using qRT-PCR with no significant changes with *SP7* gene silencing after 14 days of culture. *FABP4* gene expression was reduced only in Donor 3 with *SP7* silencing. Shapiro-Wilk test was done to test for normal distribution (α = 0.05). One-way ANOVA and Dunnett’s multiple comparison test were done for normally distributed samples. Kruskal Wallis test and Dunn’s multiple comparisons test were done on samples that were not normally distributed. Statistical significance was indicated only in transduced cultures (ScControl and shRNA-*SP7*). Data plots represent the means of 4 replicate wells unless otherwise stated. *p < 0.05, **p < 0.01, ***p < 0.001, and ****p < 0.0001.

Addition of BMP-2 to BMSC osteogenic cultures not only hastens mineralization, but also induces adipogenesis.^18,20^ The presence of lipid vacuoles in osteogenic cultures was detected in all BMSC osteogenic cultures in this study and was visualized by staining with ORO (Figure 5(a)). The lipid vacuoles in osteogenic cultures were typically spheroidal, and formed in clusters, retaining the red color of the lipophilic ORO. In addition to a clustered arrangement of lipid vacuoles, individually scattered spheroid units stained with ORO were numerous in Donor 2 ScControl cultures (Figure 5(a)). DAPI staining showed a visible reduction of nuclei in shRNA-*SP7* cultures compared to WT and ScControl cultures for Donors 1 and 2 (Figure 5(a)).^21^ When adipogenesis proceeded in osteogenic cultures, image analysis showed reduced lipid vacuole production in shRNA-*SP7* cultures compared to ScControl for all BMSC donors (Figure 5(b)). In two out of three BMSC donors, the production of lipid vacuoles was proportional to their metabolic activity (Figure 5(b)).

The expression of adipogenic gene markers (*PPARG* and *FABP4*) were also quantified in osteogenic induction cultures to determine if they were affected by *SP7* gene silencing. There were no significant changes in *PPARG* gene expression when *SP7* was silenced in adipogenic cultures, relative to controls (Figure 5(c)). *FABP4* was downregulated only for Donor 3 shRNA-*SP7* cells (Figure 5(c)).

### Effect of SP7 gene silencing on BMSC adipogenic cultures

**Figure 6.**
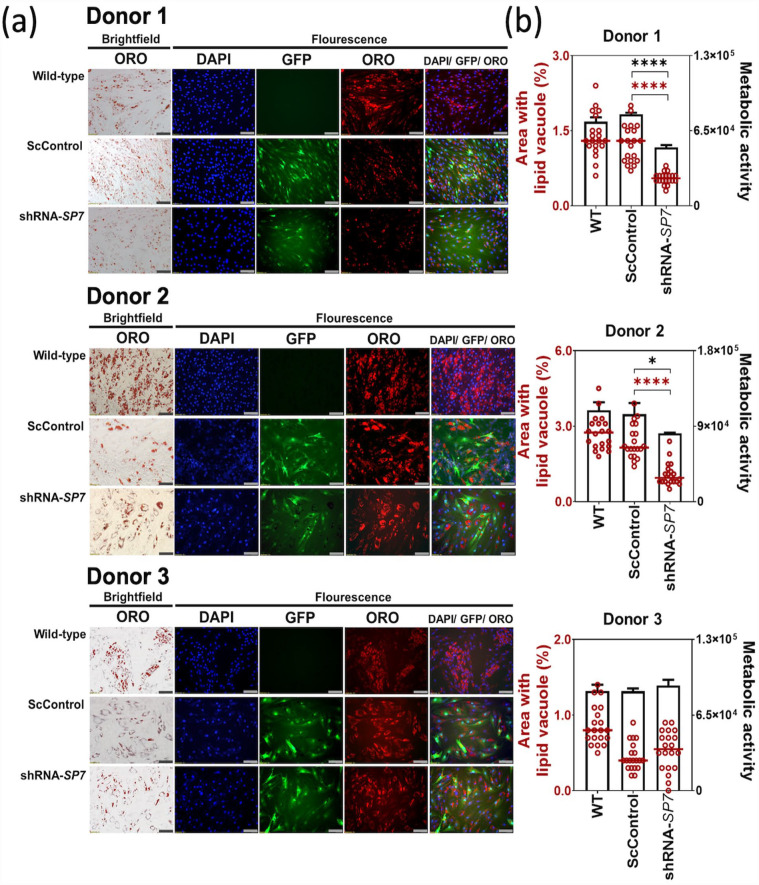
*SP7* silencing did not affect lipogenesis in BMSC adipogenic cultures. (a) Microscopic images of 14-day BMSC adipogenic cultures showed lipid vacuole formation stained with Oil Red O (ORO) viewed with brightfield microscopy, and with DAPI (blue) and ORO (red) viewed using fluorescence microscopy. GFP (green) is indicative of transduced cells in Scrambled Control and shRNA*-SP7.* Combined images were constructed using Cellsens software. (Scale bar = 100 µm). (b) Quantification of OR O-stained lipid vacuole area formed after 14 days of adipogenic culture, measured using ImageJ (*n* = 20 frames). A significant reduction in lipid vacuole formation was observed in Donor 1 and Donor 2 with *SP7* gene silencing, which can be related with metabolic activity, measured by the alamarBlue assay. Shapiro-Wilk test was done to test for normal distribution (α = 0.05). One-way ANOVA and Dunnett’s multiple comparison test were done for normally distributed samples. Kruskal Wallis test and Dunn’s multiple comparisons test were done on samples that were not normally distributed. Statistical significance was indicated only in transduced cultures (ScControl and shRNA-*SP7*). Data plots represent the means of 4 replicate wells. *p < 0.05, **p < 0.01, ***p < 0.001, and ****p < 0.0001.

Lipid vacuoles, and lipophilic staining with ORO, were consistently observed in the non-transduced WT, transduced ScControl, as well as the shRNA-SP7 samples for all BMSC donors. This indicated that *SP7* gene silencing did not inhibit lipid formation during BMSC adipogenesis (Figure 6(a)). However, quantification of the ORO-stained areas by image analysis showed reduced lipid vacuole formation in both Donor 1 and Donor 2 shRNA-*SP7* adipogenic cultures (Figure 6(b)). In all BMSC donors, the pattern of quantified lipid vacuole formation follows that of the metabolic activity (Figure 6(b)).

## Discussion

To maximize the potential utility of BMSCs as a cell source for cartilage repair, hypertrophy must be prevented or at least, minimized. Several strategies to counter hypertrophic differentiation have been attempted,^2,22^ but none have thus far been widely reproduced or translated clinically. We recently reported that *SP7* is upregulated in BMSC chondrogenic induction cultures following a single day of TGF-β1 stimulation, and that *SP7* remains upregulated even if TGF-β1 is subsequently removed from the medium.^3^ Based on this observation, and on the known role that *SP7* plays in bone development,^4–6^ we explored the impact of *SP7* silencing with shRNA on BMSC differentiation.

Initially, we determined the conditions when *SP7* is upregulated. Monolayer expanded cultures of BMSCs from three different BMSC donors in this study showed undetectable or very low *SP7* expression. *SP7* is upregulated when BMSCs are induced with an appropriate signaling molecule,^8,23^ and in this study *SP7* was significantly upregulated when cultures were chondrogenically induced with TGFβ-1 and or osteogenically induced with BMP-2. *SP7* encodes a transcription factor that acts downstream of the osteogenic master regulator, *RUNX2*.^5^ The growth factor, BMP-2, upregulates *RUNX2* expression, and RUNX2 binds directly to the *SP7* promoter to drive its expression in addition to other target genes.^24^ In the case of TGF-β1, upregulation of the *SP7* gene is prominent even after a single day of exposure during the chondrogenic induction of BMSCs.^3^ The mechanism of TGF-β1 influence on the upregulation of *SP7* in BMSCs is not yet fully understood. But like BMP-2, TGF-β1 signals through the Smad 1/5/8 signaling pathway^25^ to regulate the expression of hypertrophic markers and requires TGF-β-induced early gene 1 (Tieg1) for *SP7* gene expression.^26^ Upregulation of *SP7* with growth factors may be cell type dependent. For instance, in mouse rib-derived chondrocytes cultures, BMP-2 enhances *SP7* (*Osterix*) gene expression, but TGF-β1 does not.^23^

*SP7*-silenced BMSC microtissues responded to TGF-β1 with a promising reduction in expression of *COL10A1*, an early marker of chondrogenic hypertrophy, *IHH*, which coordinates key events for endochondral ossification, and *COL1A1* (2/3 donors), an early marker for osteoblast differentiation.^27^ However, there was a parallel reduction in the expression of important chondrogenic markers, including *COL2A1* and *ACAN*.^28^ Silencing *SP7* had a visually appreciable impact on chondrogenesis; microtissues generated from shRNA-*SP7* BMSC did not increase in size like WT and ScControl microtissues, and these tissues produced significantly less GAG. While lacunae structures were evident in cartilage-like tissue generated from WT and ScControl cells, they were less evident, even appearing to have collapsed in tissues generate from shRNA-*SP7* BMSCs. In totality, silencing *SP7* did dampen hypertrophy, but in parallel, it also significantly compromised BMSC chondrogenesis.

The role of *SP7* in embryonic and postnatal bone formation is well-documented,^5,6^ but has not been characterized for adult human BMSC osteogenesis. Osteogenic differentiation cultures in this study were supplemented with BMP-2, which upregulated *SP7* expression, and *SP7* silencing was evaluated to determine if this strategy could counter the effects of the BMP-2. Silencing of *SP7* reduced the expression of osteogenic markers, *ALPL* and *BSP*, for all BMSC donors, but only dampened mineralization for two out of three donors. The influence of *SP7* on the regulation of *ALPL* and *BSP* was reported previously using knockout mouse models or murine cell lines which behavie similarly to the human BMSCs used in this study,^6,7,9,29^ and the reduction in mineralization we observed may be attributed to the downregulation of osteogenic gene marker expression. The presence of mineralization in *SP7*-silenced Donor 3 osteogenic cultures may be due to donor variability, but it is more likely a reflection of other signaling cascades that remain intact despite *SP7* silencing. In a similar study, Zhu et al. treated BMSC, having *SP7* knockdown, with BMP-6, and observed mineralization despite downregulation of osteogenic markers (*BSP*, *OPN*, *OMD*, and *ASPN*).^30^*RUNX2* acts upstream of *SP7*^5^ and its expression was not affected by *SP7* silencing for all three BMSC donors in our study, suggesting that other *RUNX2-*target genes may be active despite *SP7* silencing. Nevertheless, *SP7* likely plays a significant role in the manifestation of BMP-2 driven osteogenesis, as BMP-2 potency is significantly blunted by *SP7* silencing. This is consistent with findings reported by Zhu et al. where *SP7* had insignificant influence on BMSC osteogenic differentiation when exogenous BMP was not added to the osteogenic medium.^30^ In totality, *SP7* has a significant influence on BMSC osteogenesis, but it does not act alone, and thus other factors can support modest osteogenesis even when *SP7* is impaired.^31^

Lipid formation increases with BMP-2 addition in BMSC osteogenic cultures.^18,20^ We also tested the capability of *SP7* silencing to affect lipogenesis as a consequence of BMP-2 induced osteogenic differentiation. In all BMSC donors, *SP7* gene silencing had minimal influence on the downregulation of *PPARG* (in all donors) and *FABP4* (2/3 donors) with the formation of lipid vacuoles still detected despite the gene silencing. The impact of *SP7* on adipogenesis is not definitive and varies depending on the cell type. In adipogenically differentiated 3T3-L1 mouse fibroblast cells, knockdown of *Sp7* enhanced *Pparg* gene expression with a consequent increase in lipid formation, and the effect was reversed with *Sp7* overexpression.^32^ In murine embryonic stem cells and murine BMSCs, *Sp7* expression did not affect *Parg* expression^33^ and lipoprotein lipase production,^29^ respectively. Lipogenesis during adipogenic differentiation of BMSCs showed similar results wherein the lipid formation proceeded even with *SP7* gene silencing. When the area stained with ORO was quantified, to estimate lipid vacuole area, lipid vacuoles were seen to be reduced for 3/3 donors in osteogenic assays and for 2/3 donors in adipogenic assays, but the reduced relative vacuole area may have been an artifact of the reduced cell numbers in these same cultures. Overall, *SP7* silencing did not appear to significantly impact BMSC adipogenesis.

In summary, the primary goal of this study was to determine if *SP7* silencing could prevent BMSC hypertrophy. The precise role of *SP7* in cartilage formation is not well understood, but reports of *SP7* gene expression detection in differentiating chondrocytes in mouse embryos,^4,5^ in adult human articular chondrocytes,^34^ and multipotential stromal cells from osteoarthritic knees^35^ suggest that it may play some role in cartilage development.^4^ If *SP7* does play a role in cartilage development, then this could provide insight into why *SP7* silencing had the negative impacted on chondrogenesis observed here. However, we reason that the significant impact *SP7* silencing has on both chondrogenic and hypertrophic differentiation cascades likely indicated that these are parallel and intertwined processes for BMSCs. This parallel process would be in contrast to fetal mesenchymal cells which are capable of sequential chondrogenic, followed by hypertrophic differentiation processes.^36^ In our earlier cited study which tracked BMSC response to TGF-β1 stimulation, we observed that chondrogenic and hypertrophic differentiation cascades appeared to played out in parallel.^3^ We also previously attempted to counter BMSC hypertrophy using a small molecule that inhibits BMP signaling.^20^ Like *SP7*, *BMP2* signaling is upregulated in BMSC following a single day of TGF-β1 stimulation, and *BMP2* signaling remains elevated even if TGF-β1 is subsequently removed from the medium. In the previous BMP-inhibition study, similar to *SP7* silencing, we observed that inhibiting BMP signaling did dampen hypertrophic differentiation, but that this resulted in a parallel dampening of chondrogenic differentiation. Previous studies which used dorsomorphin to dampen BMSC hypertrophy also observed that this resulted in a parallel obstruction in the initiation of chondrogenesis.^37^ Cumulatively, these data suggest that BMSC chondrogenic and hypertrophic differentiation are intertwined and highlight the challenge of maintaining chondrogenesis whilst simultaneously obstructing hypertrophy.

A limitation of this study is that only three BMSC donor populations were utilized, and in some cases, there was variation in outcomes between donors. For all three donors, the major hypertrophy marker, *COL10A1* gene expression, was also significantly dampened by *SP7* silencing. However, major markers of chondrogenic differentiation including GAG content, *ACAN* gene expression, and *COL2A1* gene expression were also significantly dampened by *SP7* silencing for all BMSC donors. Cumulatively, these data demonstrated that *SP7* silencing consistently resulted in dampening of both chondrogenic and hypertrophic differentiation. While these major signals were consistent, there was variation between BMSC donors with respect to secondary signals, such as *COL1A1* and *RUNX3* gene expression during chondrogenesis, and in osteogenic differentiation assays. In osteogenic assays, *ALPL* and *BSP* were significantly reduced by *SP7* silencing for all three BMSC donors, but despite a significant reduction in these major osteogenic gene pathways, one *SP7* silenced BMSC donor cell population mineralized. It may be that osteogenic pathways that are independent of SP7 may still be activated and allow BMSCs to eventually mineralize in osteogenic induction cultures. Future studies might consider using additional BMSC donors, or studies might use high-resolution tools such as RNA-Seq to better deconvolute the role *SP7* silencing has on BMSC differentiation. Additionally, especially for osteogenic differentiation cultures, it may be useful to collect more timepoints, including longer timepoints to understand if all BMSC populations eventually mineralize despite *SP7* silencing and dampened *ALPL* and *BSP* gene expression.

## Supplemental Material

sj-docx-1-tej-10.1177_20417314231177136 – Supplemental material for SP7 gene silencing dampens bone marrow stromal cell hypertrophy, but it also dampens chondrogenesis Click here for additional data file. Supplemental material, sj-docx-1-tej-10.1177_20417314231177136 for SP7 gene silencing dampens bone marrow stromal cell hypertrophy, but it also dampens chondrogenesis by Rose Ann G Franco, Eamonn McKenna, Pamela G Robey, Ross W Crawford, Michael R Doran and Kathryn Futrega in Journal of Tissue Engineering
