# Supplementary material for: SP7 gene silencing dampens bone marrow stromal cell hypertrophy, but it also dampens chondrogenesis
Source: J Tissue Eng. 2023 Jun 21;14:20417314231177136. doi: 10.1177/20417314231177136 (PMC10288420; doi:10.1177/20417314231177136)
Supplement: sj-docx-1-tej-10.1177_20417314231177136 – Supplemental material for SP7 gene silencing dampens bone marrow stromal cell hypertrophy, but it also dampens chondrogenesis [file sj-docx-1-tej-10.1177_20417314231177136.docx]

**Supplementary**

**Supplemental Table 1. Primers used in RT-PCR**

|  | **Gene** |  | **Sequence (5' - 3')** |
| --- | --- | --- | --- |
|  | *SP7* | F | TTCTGCGGCAAGAGGTTCACTC |
|  |  | R | GTGTTTGCTCAGGTGGTCGCTT |
| ***Housekeeping gene*** | |  |  |
|  | *GAPDH* | F | ATGGGGAAGGTGAAGGTCG |
|  |  | R | TAAAAGCAGCCCTGGTGACC |
| ***Chondrogenic genes*** | |  |  |
|  | *COL2A1* | F | GGCAATAGCAGGTTCACGTACA |
|  |  | R | CGATAACAGTCTTGCCCCACTT |
|  | *ACAN* | F | TCGAGGACAGCGAGGCC |
|  |  | R | TCGAGGGTGTAGCGTGTAGAGA |
|  | *SOX9* | F | ACTCCTCCTCCGGCATGAG |
|  |  | R | GCTGCACGTCGGTTTTGG |
| ***Hypertrophic genes*** | |  |  |
|  | *COL1A1* | F | CAGCCGCTTCACCTACAGC |
|  |  | R | TTTTGTATTCAATCACTGTCTTGCC |
|  | *COL10A1* | F | ACTCCCAGCACGCAGAATCCA |
|  |  | R | TGGGCCTTTTATGCCTGTGGGC |
|  | *RUNX3* | F | CAAGATGGGCGAGAACAGC |
|  |  | R | ATCACAGTCACCACCGTACC |
|  | *IHH* | F | ATGAAGGCAAGATCGCTCG |
|  |  | R | GATAGCCAGCGAGTTCAGG |
| ***Osteogenic genes*** | |  |  |
|  | *ALPL* | F | CGTGGCTAAGAATGTCATCATGTT |
|  |  | R | TGGTGGAGCTGACCCTTGA |
|  | *RUNX2* | F | GGAGTGGACGAGGCAAGAGTTT |
|  |  | R | AGCTTCTGTCTGTGCCTTCTGG |
|  | *BSP* | F | GGCCTGTGCTTTCTCAATGAA |
|  |  | R | GCCTGTACTTAAAGACCCCATTTTC |
|  | *BGLAP (OCN)* | F | GCAAAGGTGCAGCCTTTGTG |
|  |  | R | GGCTCCCAGCCATTGATACAG |
| ***Adipogenic genes*** | |  |  |
|  | *PPARG* | F | AAGACCACTCCCACTCCTTTG |
|  |  | R | GTCAGCGGACTCTGGATTCA |
|  | *FABP(4)* | F | ACGAGAGGATGATAAACTGGTGG |
|  |  | R | GCGAACTTCAGTCCAGGTCAAC |

**
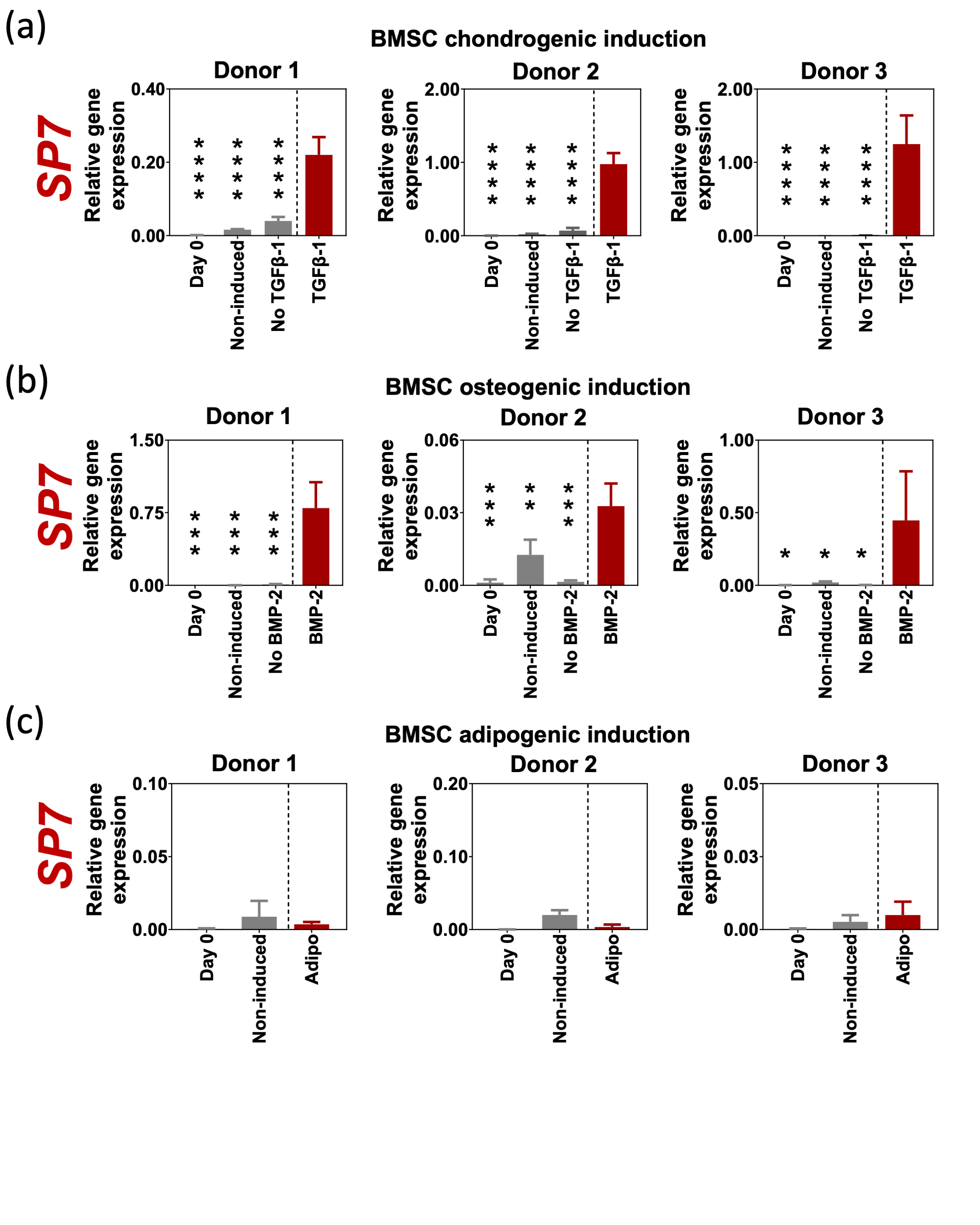
**

**Supplemental Figure 1. *SP7* gene expression in non-transduced chondrogenic, osteogenic, and adipogenic BMSC induction cultures. (a)** Elevated *SP7* gene expression was observed in chondrogenic cultures in all 3 BMSC donors after 14 days of culture with TGF-β1. Day 0 samples were BMSC monolayer cultures obtained prior to chondrogenic induction. Non-induced samples were grown in expansion medium for 14 days. No TGFβ-1 samples were grown in chondrogenic medium without TGFβ-1. TGFβ-1 samples were grown in chondrogenic medium with TGFβ-1 (10 ng/mL). Statistical significance was indicated if reported values were lower than TGF-β1 samples. **(b)** Elevated *SP7* gene expression was observed in 14-day BMSC osteogenic cultures in 3 donors. Day 0 samples were obtained prior to osteogenic induction. Non-induced samples were grown in expansion medium for 14 days. No BMP-2 samples were grown in osteogenic medium without BMP-2. BMP-2 samples were grown in chondrogenic medium with BMP-2 (100 ng/mL). Statistical significance was indicated if reported values are lower than BMP-2 samples. **(c)** *SP7* gene expression in 14-day BMSC adipogenic cultures is not significantly higher from Day 0 and 14-day non-induced monolayer samples in 3 donors. Shapiro-Wilk test was done to test for normal distribution (α = 0.05). One-way ANOVA and Dunnett’s multiple comparison test were done for normally distributed samples. Kruskal Wallis test and Dunn’s multiple comparisons test were done on samples that were not normally distributed. Data plots represent the means of 3 replicate wells. **P* < 0.05, ***P* < 0.01, ****P* < 0.001, and *****P* < 0.0001.


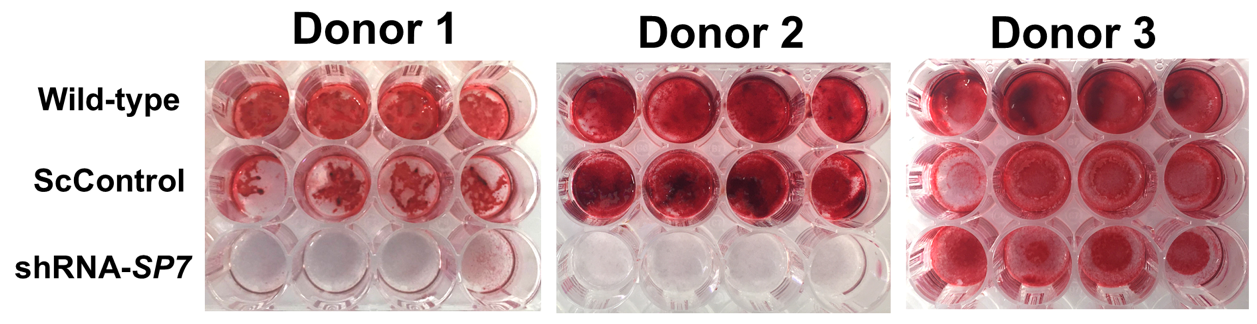


**Supplemental Figure 2.** Figure S2 includes information related to Figure 4. Alizarin Red S staining of BMSC osteogenic cultures after 14 days of osteogenic induction with BMP-2 (100 ng/mL).
